# Response to the COVID-19 Pandemic: Comparison of Strategies in Six Countries

**DOI:** 10.3389/fpubh.2021.708496

**Published:** 2021-09-30

**Authors:** Haiqian Chen, Leiyu Shi, Yuyao Zhang, Xiaohan Wang, Jun Jiao, Manfei Yang, Gang Sun

**Affiliations:** ^1^Department of Health Management, School of Health Management, Southern Medical University, Guangzhou, China; ^2^Department of Health Policy and Management, Bloomberg School of Public Health, Johns Hopkins University, Baltimore, MD, United States

**Keywords:** COVID-19, containment strategy, mitigation strategy, countries comparison, public health

## Abstract

**Objective:** This study aimed to examine the effectiveness of containment strategies and mitigation strategies to provide a reference for controlling the ongoing global spread of the pandemic.

**Methods:** We extracted publicly available data from various official websites between January 1 and December 31, 2020, summarized the strategies implemented in China, South Korea, Singapore, the United States, the United Kingdom, and France, and assessed the effectiveness of the prevention and control measures adopted by these countries with the daily new cases and mortality rate per 100,000 population.

**Results:** China, South Korea, and Singapore adopted containment strategies, which maintained a proactive approach by identifying and managing cases, tracking and isolating close contacts. China and Singapore had a similar epidemic curve and the new daily cases. As of December 31, 2020, the new daily cases of China and Singapore were below 100 with the mortality rates per 100,000 population of 0.3 and 0.5, respectively. But the new daily case of South Korea was as high as 1,029, with a mortality rate per 100,000 population of 1.8. In contrast, the United States, the United Kingdom, and France responded with mitigation strategies that focus on treating severe cases and those with underlying conditions. They had similar epidemic curves and mortality rates per 100,000 population. The United States had up to 234,133 new confirmed cases per day, and the mortality rate per 100,000 population was 107, while the United Kingdom had 56,029 new confirmed cases per day and the mortality rate per 100,000 population was 108, and France had 20,042 new cases per day, with a mortality rate per 100,000 population of 99.

**Conclusions:** China, Korea, and Singapore, which implemented strict containment measures, had significant outbreak control. Meanwhile, the successful practices in China, Singapore, and South Korea show that the containment strategies were practices that work especially at the individual level identifying and managing the infected patients and their close contacts. In the United States, the United Kingdom, and France, which implemented the mitigation policies, the effect of epidemic prevention and control was not significant that the epidemic continued or even increased epidemic relatively quickly.

## Introduction

The coronavirus disease 2019 (COVID-19) is continuing to spread worldwide. As of Feb 23, 2021, the COVID-19 outbreak has caused 112,222,860 confirmed cases and 2,483,930 death cases. The number of new cases outside China exceeded 290,000, the total of confirmed cases was more than 110 million, and the total number of death cases was more than 2.47 million (1). However, fortunately, in the past year, at least 186 countries have implemented varying degrees of restrictions on population movement to slow the spread of the severe acute respiratory syndrome coronavirus (SARS-CoV) and prevent health systems from becoming overwhelmed. These restrictions have amounted to lockdowns in 82 countries, resulting in a retreat in new cases and the mortality rate (2, 3).

In response to the COVID-19 epidemic that is ravaging the world, countries have employed various strategies for controlling the pandemic based on their different economic, cultural, and health system situations. These epidemic control strategies can be divided into two types: one type is a containment strategy, and the other is a mitigation strategy. A containment strategy focuses on disease prevention and the control of infectious diseases from three aspects: infectious sources, transmission routes, and susceptible populations (4). It aimed to break the chain of transmission through a combination of aggressive test-and-isolate policy (identify and isolate all infectious persons, including those with mild illness) and social distancing measures (5). Furthermore, the containment strategy abided by “five early's” principles (early detection, early report, early investigation, early isolation, and early treatment). The confirmed cases and suspected cases were treated in intensive until the medical observation period was complete (4).

Whereas, a mitigation strategy focuses on reducing the transmission rate, asserting that the spread of COVID-19 cannot be completely interrupted and can only be slowed when the population forms an adequate immune barrier and the intensity of the epidemic decreases to become a seasonal infection, such as influenza (4). It aimed to reduce death tolls by focusing on the medical care of severe cases while relying on social distancing to flatten the curve of epidemic impact on healthcare systems (5). Moreover, the mitigation strategy prioritizes hospitalization for severe cases or those with the underlying disease rather than early detection of all cases, isolates and treats mild cases, or screens and manages close contacts (6).

In order to compare the effects of different types of non-pharmaceutical interventions, this study selected China, South Korea, and Singapore of Asia with earlier epidemics, these countries implemented containment strategies to successfully contain COVID-19 with cases and close-contact identification and management. Meanwhile, we also selected the United States, the United Kingdom, and France, these countries implemented mitigation strategies that tried to control the COVID-19 by actively treating severe cases or those with underlying diseases but were experiencing a severe outbreak of COVID-19. We hoped that our findings would provide a policy reference for the countries experiencing the impact of the COVID-19.

## Methods

### Data Collection

The epidemiological data are extracted from official websites and updating in real-time, including the National Health Commission of the People's Republic of China, Johns Hopkins University & Medicine Coronavirus Resource Center, and Worldometer, which has synthesized data from government websites of countries (7–9). Data indicators include national population, totally confirmed cases, daily new cases, total deaths, and daily new deaths. We calculated the mortality rate per 100,000 population using the national population and the total deaths.

### Policy Information

Information on the control strategies, policies, and measures of six countries were searched from national documents and government webpages of various countries, such as media announcements and governmental decrees between January 1 and December 31, 2020. The control strategies, policies, and measures were categorized into containment and surveillance, healthcare, border control, and community and society measures.

Finally, we selected epidemiological data and policy information from January 1 to December 31, 2020, and assessed the effectiveness of the COVID-19 strategies adopted by these countries by combining the strategies of the six countries with the daily new cases and mortality rate per 100,000 population.

## Results

### The National Response to the COVID-19 Pandemic

#### China, South Korea, and Singapore Containment Strategies

China was the first country to report the COVID-19 infection. South Korea and Singapore were the following rapidly hit countries after China. At the beginning of the COVID-19 outbreak, with no immediate vaccines and antiviral medication for COVID-19, China being the epicenter of the outbreak swiftly swung into action in managing the epidemic. Typical measures include the use of existing traditional public health epidemic containment strategies of lockdown infectious areas, testing, isolation, quarantine, expanding the number of beds, physical distancing, and community containment (10).

Similarly, South Korea and Singapore, the next two hit COVID-19 outbreak countries after China, fully utilized their experience from the Middle East respiratory syndrome (MERS) outbreak in 2015 and the severe acute respiratory syndrome (SARS) outbreak in 2003, respectively, in responding to COVID-19. Based on the three core principles of openness, transparency, and creative innovation, South Korea was able to effectively implement the strategy of 3Ts of testing, tracing, and treatment (11). However, the Singapore government had constructed a three-pronged approach which includes travel, healthcare, and community measures to curb the spread of COVID-19. The major measures taken for COVID-19 in China, South Korea, and Singapore are summarized in Table 1 from containment, healthcare, border, and community and society.

**Table 1:** The major measures taken for COVID-19 in China, South Korea, and Singapore.

**Measures**	**China**	**South Korea**	**Singapore**
Containment and surveillance measures	Implementing strictly the “Four early's” measures of early detection, early reporting, early isolation, and early treatment. (1) Early detection: performing community screening, setting up temperature testing points in neighborhoods, companies, shopping malls and other public places, and conducting nucleic acid testing screening for people with clinical symptoms, close contacts of confirmed cases, and people returning from epidemic areas. (2) Early reporting: individual initiative reporting, unit uniform reporting, pharmacy discovery reporting, medical institution reporting, joint prevention, and control reporting. (3) Early isolation/quarantine: self-quarantine at home, centralized medical isolation, and centralized hospital for observation. (4) Early treatment: clearly diagnose and transfer to a designated hospital as soon as possible.	“Three Ts” measures of fast Testing, meticulous Tracing, and appropriate Treatment. (1) Fast testing, the Korean government granted emergency use authorization for testing kits which helped to build a foundation for large-scale testing. And the introduction of drive-through and walk-through screening stations for sample collection coupled with fast and aggressive testing allowed early detection of confirmed cases in communities. (2) Meticulous Tracing: the time needed for epidemiological investigations was also significantly reduced thanks to the utilization of ICT. (3) Appropriate treatment: confirmed cases are first categorized by severity for access to appropriate treatment.	(1) At healthcare facilities or through contact tracing confirmed cases were based on clinical and epidemiological criteria, and continuously update as change of the COVID-19 situation. Doctors were also allowed to test patients who are suspected for clinical or epidemiological reasons. (2) All suspected and confirmed cases were immediately isolated in hospital. Asymptomatic close contacts were required to quarantine for 14 days. Also, the government launched the “TraceTogether” APP to trace close contacts. (3) All public hospital laboratories offer PCR testing for COVID-19 to increase national diagnostic capacity.
Healthcare measures	(1) Pairing assistance, mobilizing 29 provinces to assist different cities in Hubei province. From January 24 to March 8, 2020, a total 346 medical teams and 42,600 medical personnel were mobilized to support Hubei province. (2) Makeshift hospitals, establishing Huoshenshan hospital, Leishenshan hospital, and 16 Fangcang shelter hospitals in Hubei province, these hospitals treated more than 12,000 COVID-19 patients. (3) Classifying management of “four categories of personnel”. All confirmed cases were transferred to the hospitals for centralized treatment, suspected cases, febrile cases who might be carriers, and close contacts were sent to designated venues for isolation and medical observation.	(1) Whether public hospitals or private hospitals were committed to responding to the COVID-19 outbreak. (2) Launching Community Treatment Centers (CTCs), from March 2 to March 26, 2020, a total of 3,292 patients were admitted to 17 CTCs. (3) Case categorization by severity: asymptomatic, mild, severe, and critical. Asymptomatic patients and patients with mild symptoms were isolated at Residential Treatment Centers or self-quarantine, patients with moderate symptoms were hospitalized at Dedicated Infectious Disease Hospitals, patients with severe symptoms or extremely severe symptoms were hospitalized at Government-designated Isolation Hospitals.	(1) Activating the National Center for Infectious Diseases (NCID) for isolation and treatment of confirmed cases. (2) Implementing the “Public Health Preparedness Clinics program” –activated more than 800 fever clinics to treat fever patients and provide subsidies for citizens. (3) The Big Box at Jurong Mall was transformed into a community care facility, accepting mainly mild patients for treatment and isolation. (4) Mild and undifferentiated persons were instructed to self-isolation at home. Those with persistent or worsening symptoms are advised to return to the same doctor for evaluation and referral for testing.
Border control measures	(1) In the Guidelines on Novel Coronavirus Diagnosis and Treatment emphasized on the elements of the port health quarantine, increased the epidemiological history of travel or residence in countries and regions with serious outbreaks abroad. (2) Nucleic acid testing were required to all travelers or returning residents entering from all ports of entry. They will be released from quarantine if they do not present with symptoms and are tested negative for SARS-CoV-2 after 14 days of quarantine. (3) Implementing the health declaration system for people exit and entry, strictly carrying out entry health quarantine, and suspending the entry of foreigners with valid Chinese visas and residence permits.	(1) Adopted monitoring measures such as special entry procedures and mandatory installation of a Self-Check Mobile App to keep track and monitor the health of inbound travelers after arrival. (2) Introduced mandatory COVID-19 testing and 2-week quarantine for all inbound travelers regardless of their port of departure. (3) Visa-free entry and visa-waiver programs were also suspended, with in addition to countries that had not imposed entry bans on Korean travelers. (4) In late June, the Korean government introduced country-specific restrictions, temporarily suspending visa issuance and non-scheduled flights and requiring submission of negative PCR-test results for issuing Korea-bound flight tickets.	Escalating border control measures: (1) Since Jan 3, 2020, temperature and health screening of incoming travelers from Wuhan and extended to all travelers since Jan 29, is in place at all ports of entry. (2) Since Feb 1, Singapore imposed entry restrictions on visitors from China; returning residents and long-term pass holders are subject to a 14-days quarantine. (3) Since March 24, prohibiting short-term visitors and cruise ship stops. (4) Since March 27, everyone who enters Singapore without a Stay Home Notice at a designated facility must wear an electronic tracker.
Community and social measures	(1) Lockdown infection areas: from Jan 23 to April 7, 2020, lockdown Wuhan city. Also, the different varying degrees of blockade were imposed nationwide. (2) In China, all provinces have activated the highest-level public health emergency response. Subsequently, many tourist attractions were temporarily closed, suspending nationwide tour operations and overseas group travel and free-travel operations. (3) School closures, postponed school opening or online classes, extended Spring Festival holidays or working from home to reduce population moving.	(1) No areas have been locked down. (2) Social Distancing—Isolation/Quarantine, Stay-at-home advisory, Closure of (3) Schools, Postpone School Opening or Online Classes, Restriction on using group facilities, Restriction on group events, and Curfew by district.	(1) No areas have been locked down. (2) Before April 5, 2020, the Singapore government took standing community and social measures: focused on health education, limited recreational restrictions, moratorium on large events, implementation of leave orders and home quarantine orders for different populations, temperature testing. (3) After April 5, 2020, the government introduced strict measures: suspending work, school, and working from home.

#### The United States, the United Kingdom, and France Mitigation Strategies

Compared with China, South Korea, and Singapore, where the COVID-19 infections occurred earlier, the United States, the United Kingdom, and France seemed slow to respond to the COVID-19 outbreak and preferred to adopt mitigation strategies. The aggressive measures of the US federal government could date back to a national emergency declaration on March 13, 2020. Since then, the United States has adopted a combination of “containment” and “mitigation” strategies, with multiple channels and means of response and increasing support for prevention and control.

The government did not take more measures to control the COVID-19 epidemic before mid-March, 2020. However, the British government began implementing the mitigation strategies based on the theory of “herd immunity” until the outbreak in Italy and Spain were nearly out of control in March due to the confirmed cases of Italy was exceeded 5,000 per day, and total deaths exceeded 1,000; and the confirmed cases of Spain was nearly 10,000 per day, and total deaths exceeded 1,000 (12, 13). Subsequently, the government further implemented more stringent measures, such as city lockdown, school closures, and entertainment closures to stop the virus from spreading more widely (14). Similarly, France practiced loose mitigation strategies until mid-March. The French government was alerted only when the COVID-19 epidemic was raging, with the number of confirmed cases and deaths increased dramatically. After that, a strict mandatory stay at home was imposed, and a state of national emergency was declared (15). The major measures taken for COVID-19 in the United States, the United Kingdom, and France are summarized in Table 2 from containment, healthcare, border, and community and society.

**Table 2:** The major measures taken for COVID-19 in the United States, the United Kingdom, and France.

**Measures**	**United States**	**United Kingdom**	**France**
Containment and surveillance measures	The United States had a slow start in widespread SARS-CoV-2 testing. (1) The Trump administration announced a campaign to conduct tests in retail store parking lots across the country, but this was not widely implemented. (2) The NIH launched a new rapid test development program on April 29, 2020, Rapid Acceleration of Diagnostics. (3) As of July 1, 2020, only four states are using contact tracing apps as part of their state-level strategies to control transmission. (4) As of August 2020, the FDA had granted Emergency Use Authorizations to over 200 tests for detecting current or past infection.	(1) The United Kingdom incorporated COVID-19 testing for severe acute respiratory illness (SARI) and ILI surveillances. Starting in early June, mass antibody testing was conducted. (2) Individuals with suspected mild symptoms of COVID-19 (new continuous cough, fever or anosmia) and all members of their households to self-isolate for 7 and 14 days, respectively, and call NHS111 if required. Patients with persistent and severe symptoms were advised to contact their general practitioner (GP) ] or call emergency services. (3) On May 18, 2020, the NHS Testing and Tracing Service was launched, whereby anyone in the UK with symptoms can request an antigen test via a dedicated website.	(1) French surveillance system: according to the COVID-19 surveillance protocol, physicians suspecting a COVID-19 case have to contact immediately either the emergency hotline (SAMU-Centre 15), if the patient is seeking medical attention from a general practitioner, or a referring infectious diseases specialist at hospital level. (2) Possible cases have to be hospitalized, isolated and cared for in one of the 38 French referral hospitals designated by the Ministry of Health. (3) Setting up case definition and update with the situation of the COVID-19. Contacts are traced from the date of onset of clinical symptoms in a case.
Healthcare measures	(1) Establishing temporary hospitals: the first temporary hospital in New York was completed on March 28, 2020. (2) Expanding the number of beds: on March 28, 2020, the U.S. medical ship “Mercy” docked in Los Angeles, which can provide 1,000 beds. (3) Appropriate treatment: on August 23, 2020, the FDA approved the use of plasma from recovered individuals to treat patients with severe COVID-19. (4) From early 2020, five or six operating primarily in the U.S. began vaccine research, and COVID-19 vaccine were administered from December 14.	(1) Established temporary critical care hospitals: capacity was upgraded at Belfast City Hospital in Northern Ireland, NHS Louisa Jordan was established in Scotland, temporary critical care NHS Nightingale hospitals were built across England, and the Dragon's Heart Hospital was set up in Cardiff, Wales. (2) Primary care practitioners were advised to avoid face-to-face assessment of suspected cases. Instead, patients should be immediately isolated and referred to the local health authorities *via* a hotline.	(1) Relying on the military to reinforce medical forces. A field hospital was established in the Milus region of Alsace with a total of 30 intensive care beds on March 25, 2020. Also, France activated a medical high speed train, Air Force A330 and navy helicopters to transport critically ill patients in the east to areas with less severe outbreaks. (2) Launching the White Plan and Blue Plan to coordinate all medical resources, including hospitals, clinics, and social security agencies. Also, retired health care workers and medical students have also been mobilized to join the fight against the epidemic.
Border control measures	(1) Public health screening at Major Airport on January 22, 2020, and 11 Airports added to Screening Watch List. (2) Suspension of access to the United States: beginning March 21, 2020, U.S. border crossings closed to travel other than “core essential travel.” (3) On March 13, 2020, the federal government escalated from a public health to a national emergency, and since March 16 all states had declared a state of emergency or a public health emergency.	(1) In March 2020, the UK went into lockdown. The government banned all non-essential travel. (2) Travelers entering the UK would have to self-isolate for 14 days upon arrival to help slow the spread of COVID-19. (3) From October onwards, varying levels of lockdown were imposed in England.	(1) The France government announced a lockdown period from March 17 to May 11, 2020: ban on all travel except relating to professional activity, buying essential goods, health or family reasons or brief individual exercise. (2) From March 17, France closed its borders for 30 days. The government advised long-term residents who have lived abroad to avoid international travel or return to France for the next 30 days. (3) The government addressed that France entered a second nationwide lockdown from October 30, 2020.
Community and social measures	(1) Many additional mitigation policies have been enacted at the state level: school closures, large gathering bans, non-essential business closures, stay-at-home orders, bar/restaurant limits, and primary election postponements. (2) Lockdown infection areas: on December 3, 2020, locked down the city of Los Angeles, USA. (3) Mask mandates have been implemented: as of early August, just over half of states require individuals to wear a mask in public, although in some states without a statewide mandate local authorities have mask wearing ordinances.	(1) Implementing a series of TV, radio and social media campaigns and recommendations for behavior change in the general public. (2) The stringency of containment measures escalated: the closure of non-essential services on March 16, follow by a lockdown on March 23. (3) Closures and restrictions: schools closure, non-essential activities were prohibited. Individuals were required to stay at home and work from home where possible, with only an hour of exercise, trips for food shopping and medication allowed per day, and a social distancing measure of 2 m. (4) Mask mandates have been implemented when people take public vehicles.	(1) The first nationwide lockdown: bans on gatherings, closure of most public establishments, and closure of schools and institutes of higher education. (2) Progressive lifting of lockdown restrictions: all gatherings, meetings, activities, travel and usage of public transport were required to respect social distancing rules. (3) Masks made mandatory in an extended range of public places. (4) Curfews and second national lockdown: with similar restrictions to the first national lockdown except that primary- and secondary school children can still attend school.

### Epidemiological Trends and Population Mortality Rates of COVID-19 in Six Countries

**Figure 1:**
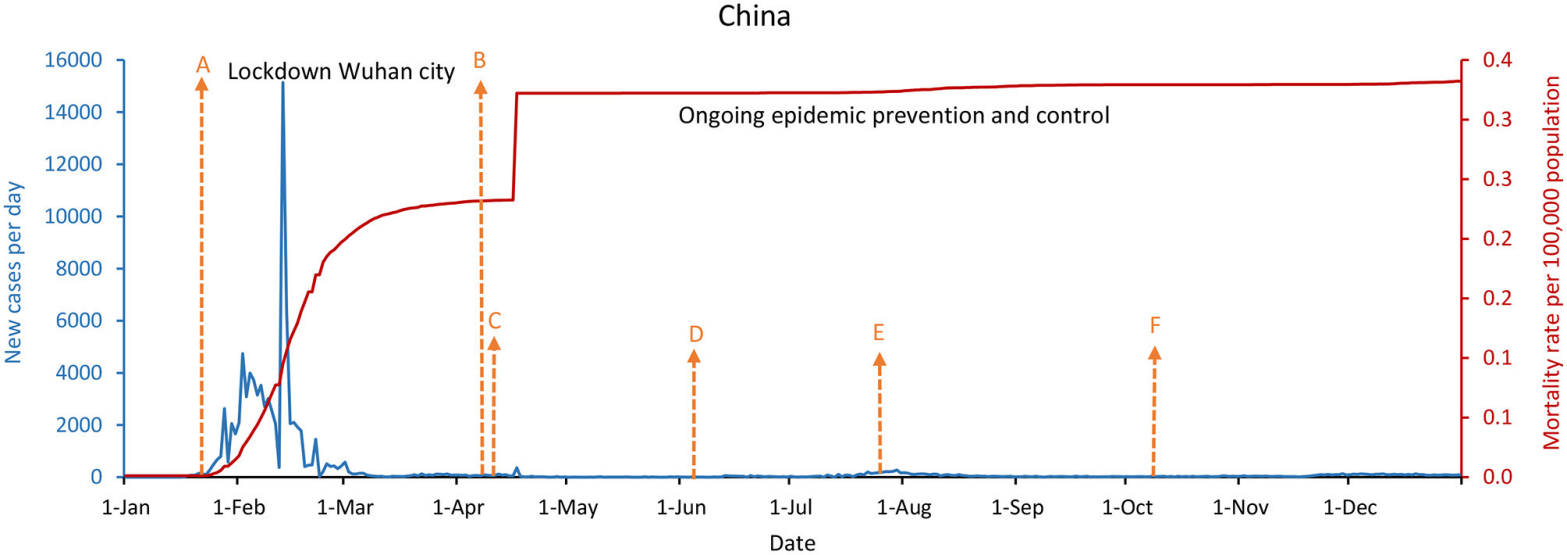
China's coronavirus disease 2019 (COVID-19) epidemic curves and population mortality rate (between January 1 and December 31, 2020). **(A)** Beginning of January 23, 2020, lockdown Wuhan city, all provinces or regions initiated a Class 1 Response Public Health Emergency. **(B)** Beginning of April 8, lifting lockdown of Wuhan city, and entering the phase of ongoing epidemic prevention and control. **(C)** On April 9, a COVID-19 cluster was detected in Heilongjiang Province. **(D)** On June 4, a COVID-19 cluster emerged at Xinfadi Market in Beijing. **(E)** On July 26, COVID-19 cases were mostly from outbreaks in Xinjiang and Liaoning. **(F)** On October 11, a COVID-19 cluster appeared in Qingdao.

**Figure 2:**
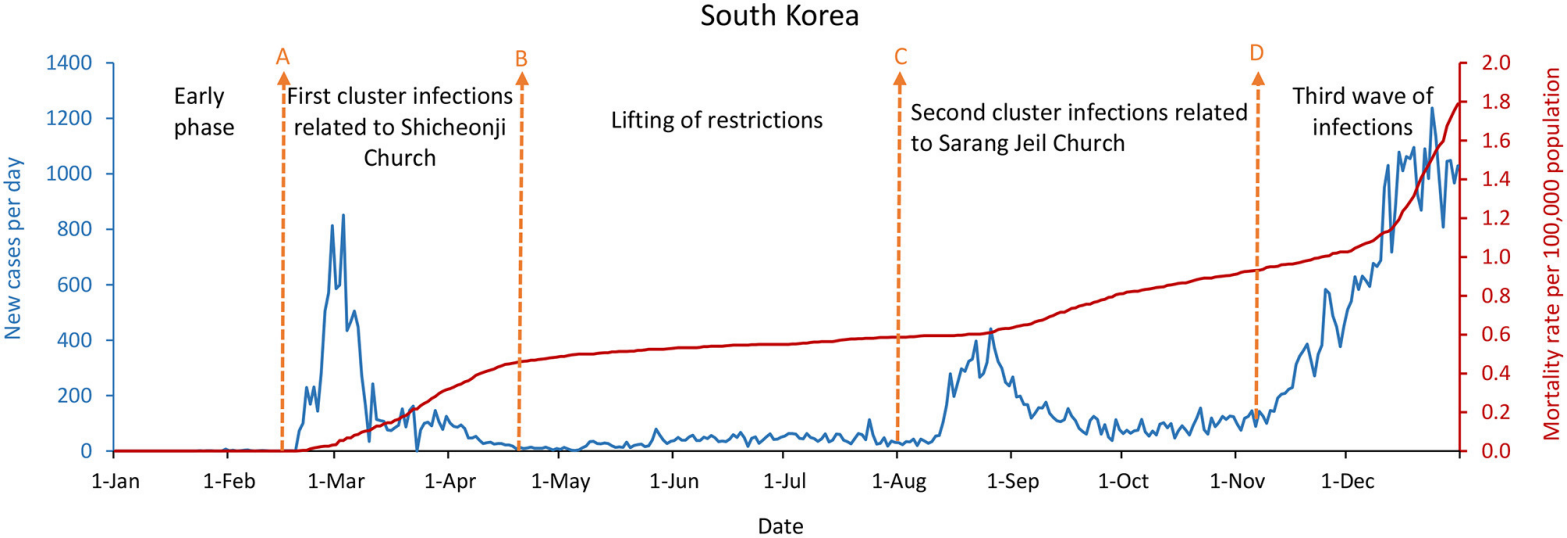
South Korea's COVID-19 epidemic curves and population mortality rate (between January 1 and December 31, 2020). **(A)** Starting on February 19, 2020, canceling mass gatherings, and various measures were taken to mass testing. **(B)** Starting on April 22, lifting restrictions of stores, restaurants, gyms, cram schools, bars, and religious services. **(C)** Starting in August, authorities ordered 12 high-risk business categories, including nightclubs, karaoke bars, buffet restaurants, and museums to cease operations; banned gatherings; imposed distancing rules; and wearing masks continued to be in place. **(D)** Starting on November 9, escalating the social distancing level.

**Figure 3:**
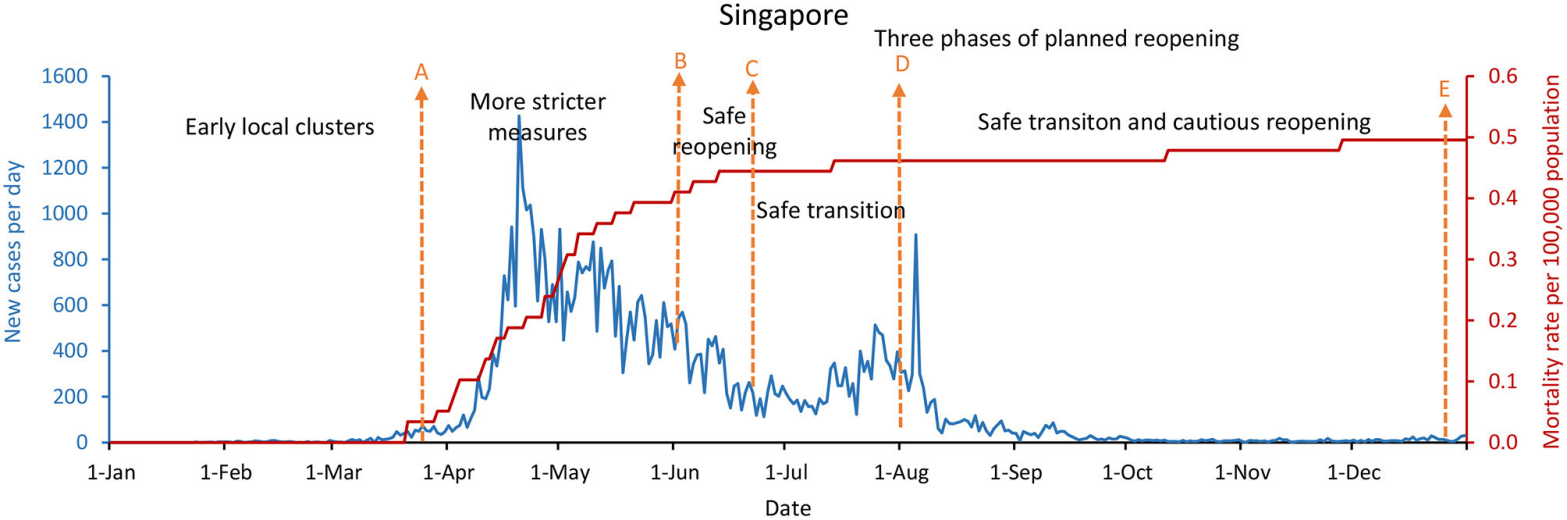
Singapore's COVID-19 epidemic curves and population mortality rate (between January 1 and December 31, 2020). **(A)** On March 24, 2020, the Multi-Ministry Task Force announced stricter measures. **(B)** Starting on June 2, relaxing measures and implementing “Safe Reopening.” **(C)** Starting on June 19, implementing “Safe Transition.” **(D)** Starting in August, continuously implementing “safe transition,” and cautious reopening. **(E)** Starting on December 28, implementing “Safe Nation.”

As shown in Figures 1–3, China, South Korea, and Singapore experienced large COVID-19 outbreaks and contained the COVID-19 outbreak with a containment strategy, especially in China and Singapore. China and Singapore had a similar epidemic curve and the number of new confirmed COVID-19 cases by December 31, 2020. In terms of mortality rate per 100,000 population, the rates of China, South Korea, and Singapore were 0.3, 1.8, and 0.5, respectively. As of December 31, 2020, especially in China and Singapore, which maintained a low mortality rate per 100,000 population no more than 1.0, new confirmed cases per day were only 87 and 30, respectively. However, new confirmed cases per day in South Korea were as high as 1,029 due to the infections linked to hospitals, nursing homes, churches, prisons, and family gatherings during the holidays.

**Figure 4:**
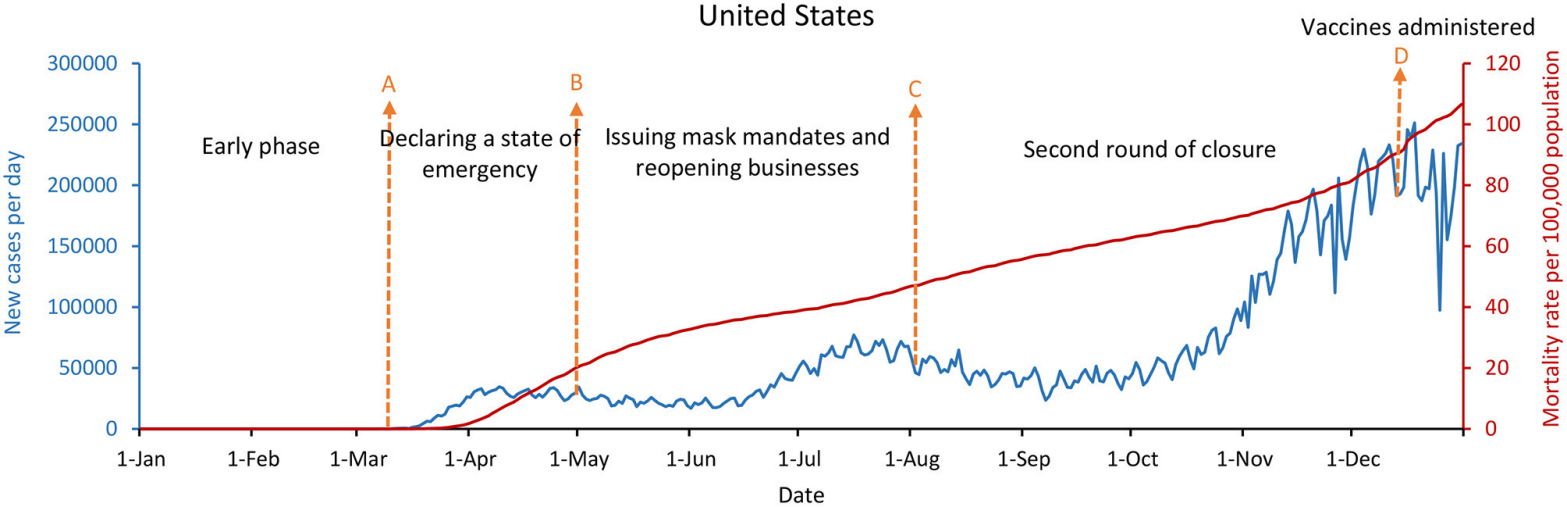
The United States' COVID-19 epidemic curves and population mortality rate (between January 1 and December 31, 2020). **(A)** By March 13, 2020, the federal government escalated from public health to a national emergency, and by March 16, all the states had declared a state of emergency or a public health emergency. **(B)** Starting in May, reopening of businesses and restaurants, and masks mandate for everyone in public spaces. **(C)** Starting in August, the second round of closure. **(D)** Starting on December 14, the first doses of the COVID-19 vaccine were administered.

**Figure 5:**
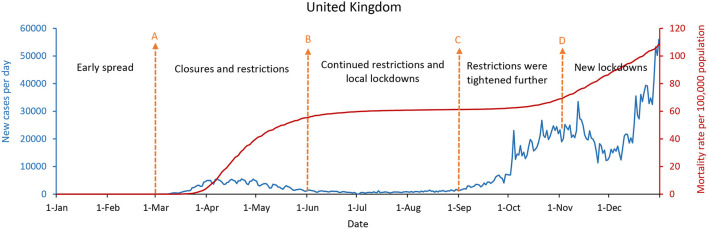
The United Kingdom's COVID-19 epidemic curves and population mortality rate (between January 1 and December 31, 2020). **(A)** Starting in March 2020, closures and restrictions. Closures and cancelations in March, lockdown continues in April, and lockdown easing begins. **(B)** Starting in June 2020, continued restrictions and local lockdowns. Requiring individuals to self-isolate for 14 days upon arrival, making face masks compulsory on all public transport, and delaying lockdown restrictions. **(C)** Starting in September, the restrictions were tightened further. **(D)** Starting in November, new lockdowns.

**Figure 6:**
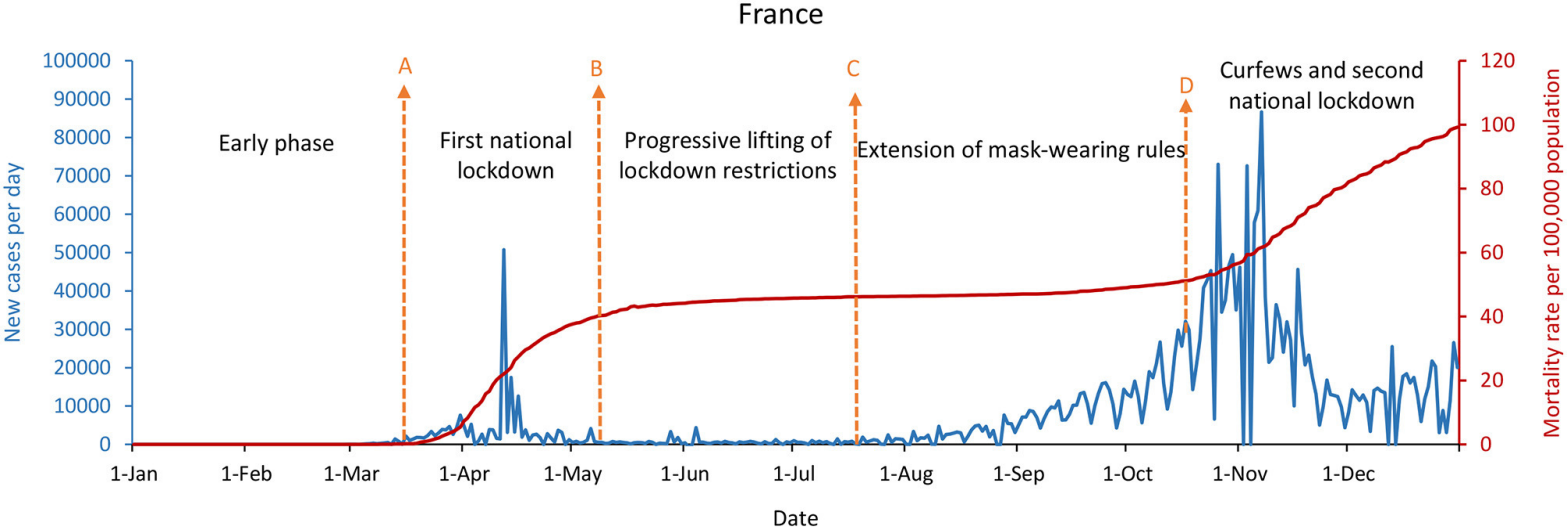
France's COVID-19 epidemic curves and population mortality rate (between January 1 and December 31, 2020). **(A)** Beginning of March 17, 2020, first national lockdown. **(B)** Beginning of May 11, progressive lifting of lockdown restrictions. **(C)** Beginning of July 20, an extension of mask-wearing rules. **(D)** Beginning of October 17, curfews and a second national lockdown.

Figures 4–6 showed that the United States, the United Kingdom, and France, which responded with a mitigation strategy when the COVID-19 pandemics emerged, had similar epidemic curves and mortality rates per 100,000 population by December 31, 2020. The daily new cases of these three countries were decreased between May and July with the mitigation strategies. However, with economic recovery and restrictions relaxing, these three countries were experiencing the second wave of the epidemic, with a doubling in daily new cases compared with the first wave. As of December 31, 2020, the United States had up to 234,133 new confirmed cases per day, and the mortality rate per 100,000 population was 107, while the United Kingdom had 56,029 new confirmed cases per day and the mortality rate per 100,000 population was 108. France had 20,042 new cases per day, with a mortality rate per 100,000 population of 99.

Whether in the new confirmed cases per day, or the mortality rate per 100,000 population, the difference is significantly remarkable between China, South Korea, and Singapore, which implemented the containment strategies, and the United States, the United Kingdom, and France, which took the mitigation strategies. Figures 2, 4–6 showed that South Korea, the United States, the United Kingdom, and France all had a similar epidemic curve by December 31, 2020. Nevertheless, South Korea had a case fatality rate of ~1% of countries adopting a mitigation strategy (South Korea: 1.8 vs. the United States: 107; United Kingdom: 108; and France: 99, by December 31, 2020).

## Discussion

There are differences in healthcare workers, health systems, health authority model, political systems, and cultural customs among China, South Korea, Singapore, the United States, the United Kingdom, and France, so their prevention and control strategies combat the COVID-19 outbreak differ. China, South Korea, and Singapore have maintained a proactive approach in responding to the COVID-19 outbreak by identifying and managing cases, tracking and isolating close contacts, and strictly restricting or controlling population movement when feasible and appropriate. Although no large-scale embargoes were implemented in Singapore and South Korea, and the outbreak rebounded in South Korea, these three countries have adopted a containment strategy based on the nature of prevention and control policies. In contrast, the United States, the United Kingdom, and France have implemented nationwide lockdown; however, these three countries focus on treating the severe cases and those with underlying conditions, and they have implemented measures that are essentially mitigation strategies.

### Containment Strategy

China's experience with SARS exposed weaknesses in the public health system and prompted a rethink of epidemic prevention policies. The government subsequently invested 6.8 billion RMB (US$850 million) to establish a new three-level network of disease control and prevention systems (16). Meanwhile, after decades of exploration and improvement, China has gradually constructed a public health system with medical institutions and medical administrative institutions (17). Wuhan experienced the problem of insufficient healthcare workers in the early stage, but the integration of the public health system and national power successfully transferred to “health care to all” (18). During combating the COVID-19 epidemic, the public health system of China mobilized the government and all sectors of society, unified command, tracked the overall situation of the epidemic, and scrambled to adapt to the development of the epidemic. For example, given the Chinese Spring Festival approaching, the national population flow would reach the peak, in order to control the continued export of infected patients in Wuhan, to avoid the nationwide spread of the epidemic, Wuhan must be locked down. After Wuhan city lockdown, responding to a dramatic increase in cases and inadequate health resources, mobilizing healthcare workers from other provinces to support Hubei, erecting Huoshenshan hospital, Leishenshan hospital, and 16 Fangcang shelter hospitals. Moreover, to interrupt the chain of transmission of the epidemic, a series of strict containment strategies were imposed in communities, screening and classifying management of “four categories of personnel,” and implementing “four early's” measures (early detection, early diagnosis, early isolation, and early treatment) to the community, even to individuals. After the battle of Wuhan, the subsequent outbreaks of sporadic epidemics and even localized clusters in Harbin of Heilongjiang, Shulan of Jilin, Xinfadi of Beijing, and Qingdao of Shandong, all proved to be the most valuable window of time for China's full-scale nucleic acid testing (19).

Similar to China, after the SARS epidemic in 2003, Singapore invested a lot of resources in improving its epidemic prevention system, establishing an interdepartmental working group pre-planning system that can be activated immediately when it encounters a public health crisis and operates in a whole-of-government manner. It has also established a public health system that includes community general clinics, public hospitals, and the National Centre for Infectious Diseases (20). Singapore, a city-state and global travel hub in Southeast Asia faced a significant risk of imported cases and implemented strict travel-related measures that all travel restrictions and quarantine orders are capped at the standard 14 days based on the COVID-19 incubation period (21, 22). Since limited community transmission emerged, Singapore implemented strict surveillance and smart tracking measures using TraceTogether, the Ministry of Health raised public awareness on the importance of personal hygiene, tracking investigation combined with early isolation, early treatment, and other means effectively control the spread of the virus in the community. With a large number of migrant workers in Singapore, there was a surge in confirmed cases in early April 2020 when multiple clusters of foreign worker dormitories were discovered. A task force was formed to contain the spread in the dormitories and ensure the welfare of the workers. The task force sealed off dormitories with infection clusters, isolated those with symptoms, and moved some workers to new accommodations. Strict sanitation, hygiene treatment, and security isolation measures were implemented. Medical support was deployed to these quarters for early and extensive testing, isolation, and treatment (22).

South Korea experienced a public health crisis caused by MERS in 2015, which exposed a weakness in the national health disaster response system. Since then, improvements have been made at all levels and throughout the public and private health sectors to protect society from the threat of emerging infectious diseases (23, 24). After 5 years, the COVID-19 pandemic occurred. Without the stringent control measures adopted by most countries, Korea was very successful in rapidly smoothing the epidemic curve in the early stages of the epidemic by scaling up testing to detect cases as early as possible (25). Such as establishing more than 600 screening sites that are capable of performing SARS-CoV-2 nucleic acid tests, including public healthcare clinics, drive-through centers, and walk-in screening sites. Other measures include school closures, locking down areas with severe outbreaks, and banning gatherings (6). In late April 2020, the daily new cases reached their lowest level (<10 cases). However, since the lifting of strict restrictions, such as keeping social distance in early May, community transmission with unknown sources of infection and influx of foreign cases have continued. In addition, a series of outbreaks occurred at several large-scale gatherings and spread to local cities (24).

### Mitigation Strategy

The United States is a wealthy country and has a well-developed healthcare system, but it has relatively poor health status and healthcare coverage and does not provide its population with the best and most equitable healthcare treatment. The US insurance system is primarily based on private employers, and individual coverage is voluntary (25). Based on these characteristics, the United States is armed with numerous high technological and biological tools to fight the COVID-19 outbreak (10). However, the initial United States response to the pandemic was otherwise slow, in terms of preparing the healthcare system, stopping other travel, and testing. Meanwhile, the leader still remained optimistic (26). With the COVID-19 cases confirmed in all 50 states of the United States, the country has begun to implement a series of mitigation measures, including all the states that had declared a state of emergency or a public health emergency, school closures, extensive gathering bans, non-essential business closures, stay-at-home orders, bar/restaurant limits, primary election postponements, and mask-wearing ordinances. Unfortunately, the lack of national leadership and a patchwork of state and local government responses but perhaps most detrimental is the division of society along partisan lines (27, 28). In addition, there is also a primary issue in the United States: the poor coordination of testing efforts and the inability to test at scale to provide comprehensive national (or even state) surveillance (25). These reasons had led to the highest number of cases and deaths in the United States, globally.

The United Kingdom has a well-established and respected universal healthcare system (NHS) that invests heavily in public health, but the shortage of personal protective equipment (PPE) and the deaths of healthcare workers in the early phases of pandemic posed a significant risk to the patients and healthcare workers (29). Meanwhile, the United Kingdom declared the COVID-19 epidemic as influenza in the early stage, emphasizing that COVID-19 was unlikely to be interrupted completely and focused mainly on treating severe cases, most of which had mild symptoms. Matters worsened when Vallance initially rejected “eye-catching measures,” such as stopping mass gatherings or closing schools. To widespread criticism, he floated an approach to “build up some degree of herd immunity” founded on an erroneous view that the vast majority of cases would be mild, such as influenza (30). With Italy, Spain, and France had taken firm public health action and was in complete lockdown, and the UK was also starting to work on preventing the disease. The policies were to be based on science, with an initial focus on containment, involving identifying people infected with SARS-CoV-2, contact tracing, and isolation of people with proven exposure (31). In addition, a package of intensive interventions was put in place including physical distancing, with a particular impact on leisure activities; workers being required to work from home where possible; shielding of both older individuals (70 years) and people in high-risk groups of all ages; school closures; and self-isolation of symptomatic individuals (32).

France benefits from its universal health insurance system, relatively large number of healthcare professionals and hospital beds, but the French system is complex, and the notoriously weak coordination between the different parts of the care system makes it more difficult for primary and social care providers and hospitals to mount a joint response. In addition, France experienced months-long protests and strikes by healthcare workers before the COVID-19 outbreak coming (33). In fact, the COVID-19 epidemic did not have a significant impact on France at the beginning of the outbreak. Subsequently, with the dramatic increase in new COVID-19 cases, France implemented strict intervention strategies in March 2020, such as implementing strict national lockdown, improving the COVID-19 detection, fully protecting the medical workforce, and strengthening research and clinical treatment methods for COVID-19. However, early dissemination of the government was intended to reassure the population that the probability of the virus spreading in France was low. Moreover, following the rapid spread of the virus in France toward the end of February, the government, totally unprepared for a pandemic (33). This was one reason for the poor control of the epidemic in France, the sharp increase in daily COVID-19 cases, and the high mortality rate per 100,000 population (as of December 31, 2020, the mortality rate per 100,000 population was 99).

### Containment vs. Mitigation Strategy

This study found that each country has implemented a series of non-pharmaceutical interventions at four levels of the epidemic: containment and surveillance, healthcare, border control, and community and society, but the effectiveness of the prevention and control measures were different among these six countries. China, South Korea, and Singapore, due to their experience with previous MERS and SARS epidemics, responding quickly, implementing strict interventions, and control the spread of the epidemic to keep the daily new cases and mortality rate per 100,000 population low. However, there are differences in the group behaviors of social people, such as community closure, home isolation, and social behavior self-discipline. In China, when a COVID-19 case was confirmed in a region, the community was immediately put under strict control or even lockdown, and large-scale nucleic acid testing of residents in the community, as well as tracing and home quarantine of close contacts. In addition, criminal detention will be imposed on those who conceal their travel and hinder the prevention and control of the epidemic. Also, people must wear masks to take public transports or to enter public places.

In Singapore, mask-wearing continues to be mandatory in public transport and all public places (34). In addition to the many violators who have been fined for not abiding by safe distances and gathering in excess of the maximum number of people, some restaurants, bars, and other businesses have been ordered to close and face fines for continuing safety violations. However, there are community cases of those who continue to go out and participate in activities after developing respiratory symptoms, and large-scale virus interdiction measures, such as those in the first wave of the outbreak have been relaxed (35). Furthermore, some users of TraceTogether even switched off their apps or left their tokens at home in protest (34). In South Korea, under the revised anti-infectious disease law, violators can face up to a year in prison, a 10 million won fine, or in the case of foreign passport holders deportation. However, it was only in May 2020 that the Seoul government began requiring people to wear masks on public transport and in taxis, and the weak awareness of the public not to comply with the quarantine regulations has caused mass cluster infections (36).

In contrast, the United States, the United Kingdom, and France, due to their lenient approach at the beginning of the epidemic, made the subsequent fight against the epidemic more difficult. Although a series of non-pharmaceutical interventions were implemented, and these countries have initiated vaccination programs for COVID-19, the results seem to be less than satisfactory. Of course, there are some reasons why implementing a strict containment strategy is simply not possible in the United States, the United Kingdom, and France. There are at least two reasons for this. The primary reason is that these are homes of intense liberal democratic norms, and the government cannot simply impose any type of lockdown. In Sweden, it is even constitutionally forbidden to impose lockdown unless there is a war (37). There were many violent protests, and people were even beaten or killed in the United States and France over simply mask mandates during the COVID-19 pandemic (38–40). Also, the United States is a federal system, and the United Kingdom is actually many countries combined into one, meaning that it is not possible for the central government to take over all decisions for the lower-level political units.

This study compared prevention and control strategies among China, Singapore, South Korea, the United States, the United Kingdom, and France, and examined the effectiveness of containment strategies and mitigation strategies. However, this study also has limitations that need to be considered. Other studies should be developed in order to confirm what has been achieved. Such as we can further work on the population-based epidemiological studies, respectively, in these six countries to improve non-pharmaceutical interventions.

## Conclusion

Based on this study it seems that China, Korea, and Singapore, which implemented strict containment measures, had significant outbreak control. In the United States, the United Kingdom, and France, which implemented mitigation policies, the effect of epidemic prevention and control was not significant that the epidemic continued or even increased relatively quickly. However, until the vaccine is globally available and effective, countries still need to address the current COVID-19 epidemic with non-pharmacological measures to avoid further damage. Meanwhile, the successful practices in China, Singapore, and South Korea show that containment strategies were practices that work especially at the individual level identifying and managing infected patients and their close contacts.

## Data Availability Statement

The raw data supporting the conclusions of this article will be made available by the authors, without undue reservation.

## Author Contributions

HC and GS conceived the paper and study guarantors. HC, YZ, XW, MY, and JJ collected the data. HC drafted the manuscript. LS, YZ, XW, MY, and JJ revised the manuscript. GS contributed to the critical revision of the manuscript for important intellectual content and approved the final version of the manuscript. All authors have read and approved the final manuscript.

## Funding

This work was supported by the National Social Science Fund of China (no. 16BGL184).

## Conflict of Interest

The authors declare that the research was conducted in the absence of any commercial or financial relationships that could be construed as a potential conflict of interest.

## Publisher's Note

All claims expressed in this article are solely those of the authors and do not necessarily represent those of their affiliated organizations, or those of the publisher, the editors and the reviewers. Any product that may be evaluated in this article, or claim that may be made by its manufacturer, is not guaranteed or endorsed by the publisher.
